# Magnetic Microwires with Unique Combination of Magnetic Properties Suitable for Various Magnetic Sensor Applications

**DOI:** 10.3390/s20247203

**Published:** 2020-12-16

**Authors:** Paula Corte-Leon, Valentina Zhukova, Alexandr Chizhik, Juan Maria Blanco, Mihail Ipatov, Lorena Gonzalez-Legarreta, Arcady Zhukov

**Affiliations:** 1 Department Advanced Polymers and Materials: Physics, Chemistry and Technology, Faculty of Chemistry, University of Basque Country, UPV/EHU, 20018 San Sebastian, Spain; paula.corte@ehu.eus (P.C.-L.); valentina.zhukova@ehu.es (V.Z.); oleksandr.chyzhyk@ehu.es (A.C.); mihail.ipatov@ehu.es (M.I.); lorena.glegarreta@gmail.com (L.G.-L.); 2 Departamento de Física Aplicada, EIG, Basque Country University, Universidad del País Vasco/Euskal Herriko Unibersitatea, UPV/EHU, 20018 San Sebastian, Spain; juanmaria.blanco@ehu.es; 3 Departamento QUIPRE, Inorganic Chemistry-University of Cantabria, Nanomedice-IDIVAL, Avda. de Los Castros 46, 39005 Santander, Spain; 4 IKERBASQUE, Basque Foundation for Science, 48011 Bilbao, Spain

**Keywords:** magnetic microwires, magnetic sensors, giant magnetoimpedance, domain wall propagation, magnetostriction coefficient, post-processing, magnetic anisotropy

## Abstract

There is a pressing demand to improve the performance of cost-effective soft magnetic materials for use in high performance sensors and devices. Giant Magneto-impedance effect (GMI), or fast single domain wall (DW) propagation can be observed in properly processed magnetic microwires. In this paper we have identified the routes to obtain microwires with unique combination of magnetic properties allowing observation of fast and single DW propagation and GMI effect in the same microwire. By modifying the annealing conditions, we have found the appropriate regimes allowing achievement of the highest GMI ratio and the fastest DW dynamics. The observed experimental results are discussed considering the radial distribution of magnetic anisotropy and the correlation of GMI effect, and DW dynamics with bulk and surface magnetization processes. Studies of both Fe- and Co-rich microwires, using the magneto-optical Kerr effect, MOKE, provide information on the magnetic structure in the outer shell of microwires. We have demonstrated the existence of the spiral helical structure in both studied microwires. At the same time, torsion mechanical stresses induce helical bistability in the same microwires, which allow us to consider these microwires as materials suitable for sensors based on the large Barkhausen jump.

## 1. Introduction

Recent advances in physical sensors have been greatly influenced by the development of novel functional materials with advanced physical properties. Among physical sensors, magnetic sensors play a relevant role due to the increasing demand and important applications in various industrial sectors such as microelectronics, security and electronic surveillance, automobile, aerospace and aircraft industries, energy-efficient refrigerators, medicine, home entertainment, energy harvesting and conversion, informatics, electrical engineering, magnetic recording, among others [1,2]. An essential part of magnetic sensors is a suitable magnetic material [1]. The performance of magnetic sensors is greatly influenced by the combination of physical properties of magnetic materials [2,3,4].

Among various families of magnetic materials, a family of soft magnetic materials with amorphous structure presents several important advantages [1,2,3,4,5]. As a rule, amorphous materials present superior excellent magnetic softness, such as low coercivity, high magnetic permeability and low core losses [1,5,6,7,8]. Such excellent magnetic softness of amorphous materials is commonly attributed to the glassy-like structure characterized by the absence of atomic long-range order and hence lack of defects typical for crystalline materials, i.e., grain boundaries, dislocations, twins, etc [5,6,7,8]. It is worth mentioning that similar magnetic softness can be also achieved in nanocrystalline materials [6,7]. However, amorphous magnetic materials additionally present superior mechanical properties [9,10,11,12,13]. A clear correlation of crystallization of amorphous materials and mechanical properties (i.e., tensile yield) shows the deterioration of mechanical properties upon the devitrification of amorphous microwires [13]. Consequently, the use of amorphous materials allows the development of robust magnetic devices and even magnetoelastic sensors [14,15,16,17,18,19,20,21].

Additionally, the common feature of different families of amorphous materials is that the fabrication process involves rapid melt quenching and hence, is fast and inexpensive [5,6,7,8,9,10,11,12,13].

The cylindrical geometry of amorphous wires is the most suitable for realization of either the Giant magnetoimpedance (GMI) effect [14,17,18,19,22,23,24,25] or magnetic bistability, associated with a single and large Barkhausen jump [5,26,27,28].

The origin of the GMI effect is satisfactorily explained from the viewpoint of classical electrodynamics, considering the influence of the applied magnetic field on the penetration depth, *δ*, of the AC current flowing through a soft magnetic conductor [22,23,24,25]. The DC applied magnetic field affects *δ* through the circular permeability, *μ_φ_*, which leads to a change in the impedance, *Z* [22,23,24,25,29]. Accordingly, a high circumferential magnetic permeability is essentially relevant for the GMI effect observation [22,23,24,25]. The highest GMI effect is observed in Co-based amorphous magnetic wires with low magnetostriction coefficient, *λ_s_*, and specific domain structure [26,30,31].

On the other hand, the first observations of the domain walls (DWs) propagation were also realized in magnetic wires [32]. The aforementioned magnetic bistability associated with the remagnetization process through a single and large Barkhausen jump can be realized in various families of amorphous wires [6,26,27,28]. This peculiar remagnetization process, in which a demagnetized state cannot be achieved, has been reported for magnetostrictive amorphous wires (i.e., for wires with either positive or negative, *λ_s_*) [26,27,28,33,34,35]. The magnetization switching between the two remanent states runs by ultra-fast DW propagation, and therefore such wires exhibit perfectly squared hysteresis loops. The DW velocity, usually evaluated by a system of several pick-up coils placed along the wire, usually exceeds 1 km/s [33,35,36,37].

There are several exciting applications of magnetically bistable wires, like magnetic tags, racetrack memories, magnetic logics, magnetic and magnetoelastic sensors [26,38,39,40].

Amorphous wires can be produced by various methods involving rapid melt quenching if the quenching rate for a given chemical composition of the alloys is above critical [7,41].

Different magnetic wires fabrication technologies involving melt quenching provide a vast variety of geometries. The following techniques allow the preparation of amorphous magnetic wires:

- the so called “in-rotating water” method, consisting of the rapid quenching of the molten metallic alloy into the rotating water layer, basically allows amorphous wires to be prepared with diameters typically of 80–200 μm up to 100 m long [12,17,27].
- the so called “melt extraction” technique, allows fabrication of amorphous wires with diameters of 30–60 μm and a typical length of about 10 m [42,43]. One of the disadvantages of melt extracted wires is the imperfect cylindrical shape.
- the so called Taylor-Ulitovsky technique (also referred in several publications as quenching-and-drawing method) prepares the amorphous glass-coated wires with metallic nucleus diameters ranging from 200 nm [44] up to 100 μm [45], a glass-coating with a thickness of 0.5–20 μm and up to 10 km long [41,44,45,46].

The preparation method consists in the simultaneous rapid solidification of a metallic alloy coated by an insulating and flexible glass-coating [41,46]. The main advantages of this preparation method are a wide range of metallic nucleus diameters, as well as the presence of glass- coating. The use of magnetic materials with improved mechanical and corrosive properties and biocompatibility allows the development of reliable, inexpensive and durable sensors and devices. Although the glass-coating is a source of increased internal stresses, mainly arising from the difference in the thermal expansion coefficients of the metallic alloy and the glass [46,47,48], flexible, insulating, and biocompatible glass-coating is useful for novel applications, including biomedicine or smart composites with microwire inclusions for non-destructive monitoring of external stimuli (stresses, temperature) [49,50,51,52,53,54]. Additionally, Taylor-Ulitovsky technique is the unique method for preparation of submicrometric amorphous wires, potentially suitable for magnetic logic and memory applications involving controllable DW propagation [38,39].

Arrays of cylindrical nanowires with diameters between 15 and 200 nm and length up to 50 mm can be prepared also by electrodeposition into self-assembled pores in alumina membranes as templates [55]. Individual cylindrical magnetic nanowires can be obtained after released from inside the template where they are electrolytically grown [56]. However, such nanowires are crystalline, and thus, magnetocrystalline anisotropy considerably affects their magnetic properties.

It is worth mentioning that crystalline Wiegand wires from Vicalloy (vanadium-iron-cobalt) can be also prepared by a complex technique, including cold-working the wire through a combination of stretching and twisting followed by heat treating [57].

In terms of properties (GMI effect and DW propagation), there are specific requirements for optimizing each of them. Therefore, for the GMI effect, magnetic softness (high magnetic permeability) and sufficiently “thick” diameters (above minimum penetration depth, estimated as about 0.3 μm [58]) are required. To observe fast magnetization switching, the hysteresis loops must present a rectangular shape. Additionally, to achieve a high DW velocity, the damping coefficient must be as small as possible, that can be achieved by minimizing the magnetic anisotropy constant that can be realized in amorphous or nanocrystalline wires [35,36,37]. For magnetic tag applications, the magnetic response must be as high as possible [40].

Most of the aforementioned properties can be obtained in glass-coated amorphous microwires. Thus, typically spontaneous magnetic bistability is observed in Fe-based microwires, which have low circumferential magnetic permeability owing to specific domain structure consisting of an inner axially magnetized single domain and an outer shell with radial magnetization direction [35]. Such Fe-based microwires, thus, usually present low GMI effect. In contrast, the highest GMI effect is observed in Co-rich microwires which present linear hysteresis loops, and hence, single DW propagation in such microwires cannot be observed [30,31].

The development of novel glass-coated microwires, which can present a unique combination of properties: High GMI effect as well as fast DW propagation is therefore challenging and relevant topic of application oriented research.

Several post-processing techniques have been reported recently for tailoring magnetic properties of magnetic microwires. In particular, various routes have been proposed to improve the GMI effect in Fe-rich microwires, exhibiting rectangular hysteresis loops and a rather low GMI effect in as-prepared state on one side, and allowing one to observe fast DW propagation in Co-rich microwires on the other side [35,59,60,61,62,63,64].

In this paper we overview the routes allowing to realize the GMI effect and fast DW propagation in the same microwire, and present new insights on magnetization reversal mechanism in microwires exhibiting both GMI effect and fast DW propagation.

## 2. Materials and Methods

### 2.1. Preparation, Structure and Morhpology Control

Fe- and Co- rich glass-coated amorphous microwires have been prepared by Taylor-Ulitovsky preparation method, described in details elsewhere (the compositions and diameters of microwires are provided in Table 1) [41,46].

The crystallization temperatures, *T_cr_*, have been evaluated by the Differential Scanning Calorimetry (DSC) method using a 204 F1 Netzsch calorimeter (Netzsch Co, Selb, Germany) at a heating rate of 10 K/min. The amorphous structure of the samples has been confirmed by the X-ray Diffraction (XRD) using the Bruker (D8 Advance) X-ray diffractometer with Cu K_α_ (λ = 1.54 Å) radiation. A broad halo typical for amorphous materials is observed in all as-prepared or annealed microwires.

**Figure 1:**
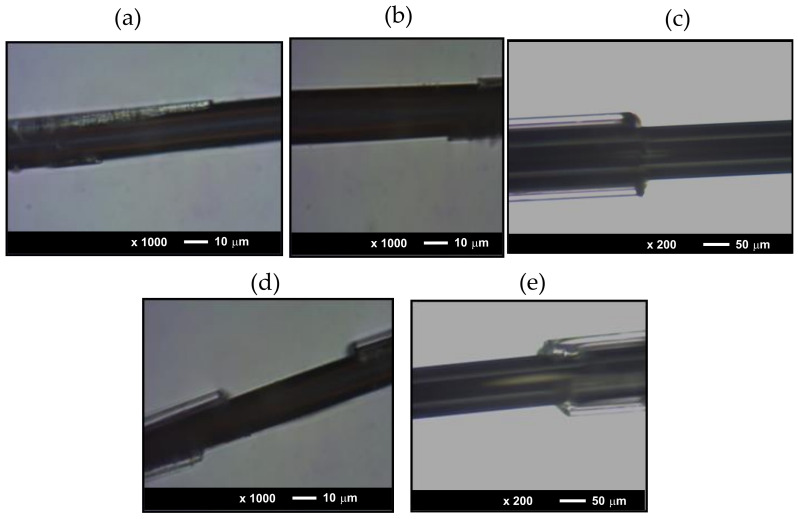
Images of studied samples 1–5 (**a**–**e**) respectively. The glass coating has been removed intentionally.

The morphology of the samples has been evaluated using Carl Zeiss -Axio Scope A1 microscope. As can be observed from Figure 1, all the studied microwires present perfectly cylindrical geometries of the metallic nucleus and rather uniform glass-coating.

We studied as-prepared and annealed microwires. Annealing was carried out in a conventional furnace at a temperature, *T_ann_*, typically below *T_cr_* (between 200 °C and 550 °C) for an annealing time, *t_ann_*, typically of 60 min. These annealing conditions preserve the good mechanical properties, typical of amorphous materials. In the case of stress-annealing, tensile stress was applied during annealing and slow cooling of the microwire inside the furnace. The stress value, *σ_m_*, was estimated considering different Young′s modulus of metal and glass as described elsewhere [61,62,63]. The *σ_m_* -value was below 500 MPa.

Considering the possible influence of surface defects and inhomogeneities, spontaneously distributed along microwires [36], we measured all properties in the same samples. Therefore, all measurements have been performed on the as-prepared sample, and then the same samples have been post-processed.

### 2.2. Magnetic and GMI Characterization

The hysteresis loops were measured using the fluxmetric method described in details earlier [65]. To compare samples of different chemical compositions (and, hence, different saturation magnetization) and annealed under different conditions, hysteresis loops were plotted as the dependence of the normalized magnetization, *M/M_0_*, on the magnetic field *H,* where *M* is the magnetic moment at a given magnetic field, and *M_0_* is the magnetic moment at the maximum magnetic field amplitude *H_m_*.

The impedance, *Z*, was evaluated from the reflection coefficient, S_11_, using a vector network analyzer and a micro-strip sample holder, as described elsewhere [66].

We evaluated the magnetic field dependencies of the GMI ratio, *ΔZ/Z,* defined as [22,23,24,25],

*ΔZ*/*Z* = [*Z*(*H*) − *Z*(*Hmax*)]/*Z*(*Hmax*) (1)

where *H_max_* is the maximum applied DC magnetic field. A uniform magnetic field, *H*, was created by a sufficiently long solenoid.

Maximum GMI ratio, ΔZ/Z_m*ax*_*,* obtained from *ΔZ/Z(H)* dependencies as a maximum *ΔZ/Z* –value can be useful for comparison of the samples, either of different chemical compositions or subjected to different post-processing (annealing) [62,63,64,65,66].

The DW velocity was evaluated using a modified Sixtus-Tonks set-up in which three pick-up coils are placed coaxially inside a long solenoid magnetizing 10 cm long microwire, as described elsewhere [35,36,37]. One of the microwire ends is placed outside the solenoid, which activates the DW propagation from the opposite microwire end. The DW velocity, *v*, can be evaluated as [35,36,37],

(2) $$v\;=\;\frac{l}{\Delta t}$$

where *l* is the distance between the pick-up coils and *Δt* is the time between the voltage peaks measured by the oscilloscope. The above-described modified setup with three pick–up coils elucidates the contribution of multiple DW nucleation at defects, evidenced by deviations from the linear *v*(*H*) dependencies in the high-field region [35,36,67].

The study of the magnetization reversal in the surface of microwires was performed by means of a high-resolution, wide-field, optical polarizing microscope based on commercial one (Carl Zeiss) working in reflective mode using the longitudinal magneto-optical Kerr effect (MOKE) configuration [68,69]. The hysteresis loops were obtained from the MOKE intensity for different values of the external magnetic field applied in the axial direction (*H_AX_*) as a result of the MOKE image processing. A mechanical torsion was applied to the studied microwire, where one of the wire edges was fixed mechanically and the other was stressed. Surface treatment and contrast enhancement with anti-reflective coatings have not been used because modern polarizing microscopy, used in the MOKE technique and digital image processing provides sufficient contrast of magnetic domains.

## 3. Results and Discussion

**Figure 2:**
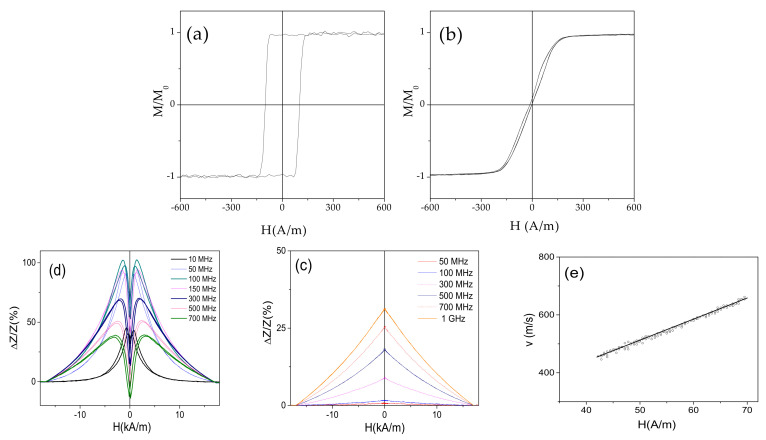
Hysteresis loops of magnetic microwires Fe_75_B_9_Si_12_C_4_ with positive (**a**) and Fe_3.6_Co_69.2_Ni_1_B_12.5_Si_11_Mo_1.5_C_1.2_ with nearly-zero (**b**) *λ_s_* values, *ΔZ/Z*(*H*) dependencies of as-prepared Fe_75_B_9_Si_12_C_4_ (**c**) and Fe_3.6_Co_69.2_Ni_1_B_12.5_Si_11_Mo_1.5_C_1.2_ (**d**) microwires and *v*(H) dependence of Fe_75_B_9_Si_12_C_4_ microwire (**e**).

The starting point for our studies are as-prepared Co-rich (Co_69.2_Fe_3.6_Ni_1_B_12.5_Si_11_C_1.2_Mo_1.5_) and Fe-rich (Fe_75_B_9_Si_12_C_4_) microwires. As shown in Figure 2a, Fe_75_B_9_Si_12_C_4_ microwire has perfectly rectangular hysteresis loop. Accordingly, GMI ratio of Fe_75_B_9_Si_12_C_4_ microwire is rather low (Figure 2c). Although, a single DW propagation with v ≈ 650 m/s is observed in Fe_75_B_9_Si_12_C_4_ microwire (Figure 2e). In contrast, linear hysteresis loop with low coercivity, *H_c_* ≈5 A/m (Figure 2b) and an order of magnitude larger maximum GMI ratio, *ΔZ/Z_max_* ≈100% (see Figure 2c) are observed in as-prepared Co_69.2_Fe_3.6_Ni_1_B_12.5_Si_11_C_1.2_Mo_1.5_ microwires. It is not possible to observe single DW propagation in as-prepared Co_69.2_Fe_3.6_Ni_1_B_12.5_Si_11_C_1.2_Mo_1.5_ microwire, owing to inclined shape of hysteresis loop typical for magnetization rotation remagetization mechanism.

Below we will focus on the description of the post-processing of Co- and Fe-rich microwires allowing to observe the GMI effect and fast DW propagation in the same microwire.

### 3.1. Tailoring of GMI Effect and DW Dynamics in Co-Rich Microwires

**Figure 3:**
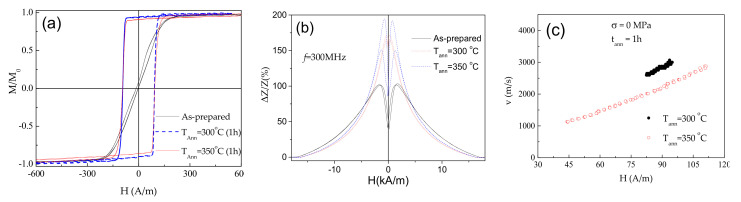
Effect of annealing on hysteresis loops (**a**), ΔZ/Z(H) dependencies measured at 300 MHz (**b**) and on *v*(H) dependencies (**c**) of annealed at *T_ann_* = 300 °C and 350 °C Co_69.2_Fe_3.6_Ni_1_B_12.5_Si_11_C_1.2_Mo_1.5_ microwires.

Similarly, to recently reported elsewhere for Co-rich microwires, annealed Co_69.2_Fe_3.6_Ni_1_B_12.5_Si_11_C_1.2_Mo_1.5_ microwire exhibits perfectly rectangular hysteresis loops (see Figure 3a). Observed magnetic hardening is previously explained, considering either internal stresses relaxation and related modification of domain structure (inner axially magnetized core volume rising) [58] or dependence of magnetostriction coefficient on internal stresses [60]. Although, usually GMI ratio in magnetic microwires with rectangular hysteresis loops is low [58,62], in the present case, a remarkable GMI ratio improvement is observed upon annealing (see Figure 3b). All Co_69.2_Fe_3.6_Ni_1_B_12.5_Si_11_C_1.2_Mo_1.5_ microwires (as-prepared and annealed) present double-peak *ΔZ/Z(H)* dependencies (see Figure 3b). However, the field values, corresponding to the maximum on *ΔZ/Z(H)* dependencies, *H_m_*, in both annealed Co_69.2_Fe_3.6_Ni_1_B_12.5_Si_11_C_1.2_Mo_1.5_ microwires are lower than that for as-prepared microwire. As discussed elsewhere, the GMI ratio value as well as the peculiarities of *ΔZ/Z(H)* dependence are determined by the type of magnetic anisotropy [70,71].

Accordingly, from Figure 3a,b we can assume that annealing allows modification of magnetic anisotropy in studied Co_69.2_Fe_3.6_Ni_1_B_12.5_Si_11_C_1.2_Mo_1.5_ microwire.

Additionally, single DW propagation with DW velocity up to 3 km/s is observed in annealed Co_69.2_Fe_3.6_Ni_1_B_12.5_Si_11_C_1.2_Mo_1.5_ microwires (see Figure 3c).

The observed *v(H)* dependencies can be described as linear, which were previously attributed to the viscous DW propagation regime given as [62,67],

*ν* = *S* (*H* − *H_0_*) (3)

where *S* is the DW mobility and *H_0_*-critical propagation field, below which the DW propagation is not observed.

**Figure 4:**
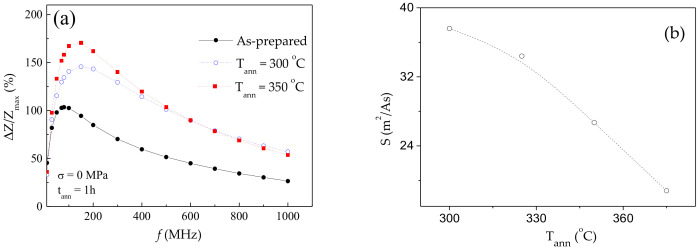
*ΔZ/Z_max_(f)* dependence of as-prepared and annealed at *T_ann_* = 300 °C and *T_ann_* = 350 °C Co_69.2_Fe_3.6_Ni_1_B_12.5_Si_11_C_1.2_Mo_1.5_ microwires (**a**) and dependence of DW mobility, *S*, on annealing temperature, *T_ann_* (**b**).

Both, GMI effect and DW mobility are affected by various factors. Thus, maximum GMI ratio, is affected by frequency, *f*, radial distribution of magnetic anisotropy (and hence, by the annealing conditions) and wire diameter, among others [62,63,64,65,71,72,73]. Accordingly, *ΔZ/Z_max_* (*f*) dependencies for as-prepared and annealed Co_69.2_Fe_3.6_Ni_1_B_12.5_Si_11_C_1.2_Mo_1.5_ microwires are provided in Figure 4a. As can be observed, both annealed microwires exhibit higher *ΔZ/Z_max_* –values in a whole frequency range. Additionally, the optimum *f*-value (at which the highest *ΔZ/Z_max_* -values are achieved) in both annealed microwires is shifted toward higher frequencies (from 100 MHz to 200 MHz).

As observed from Figure 4b, annealed Co_69.2_Fe_3.6_Ni_1_B_12.5_Si_11_C_1.2_Mo_1.5_ microwires exhibit rather high *S*-values (up to about 37 m^2^/As), however a decrease in *S*-values rising the *T_ann_* is observed.

**Figure 5:**
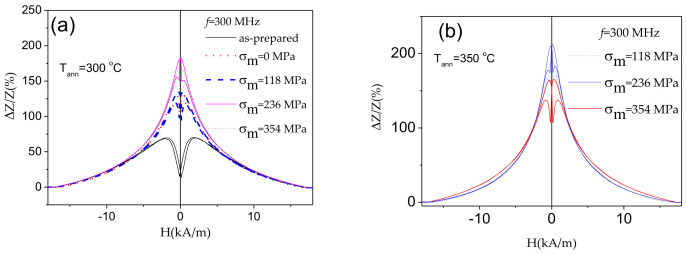
*ΔZ/Z(H)* dependencies of as-prepared and stress-annealed at different *σ_m_* at *T_ann_* = 300 °C (**a**) and *T_ann_* = 350 °C (**b**) Co_69.2_Fe_3.6_Ni_1_B_12.5_Si_11_C_1.2_Mo_1.5_ microwires measured at 300 MHz.

As recently reported elsewhere [61,62,63,64,65], stress-annealing is an effective tool allowing tuning of the magnetic anisotropy and, under appropriate conditions, to improve further the GMI effect. Accordingly, as can be observed from Figure 5a,b, stress-annealed Co_69.2_Fe_3.6_Ni_1_B_12.5_Si_11_C_1.2_Mo_1.5_ microwires exhibit even higher GMI ratio.

**Figure 6:**
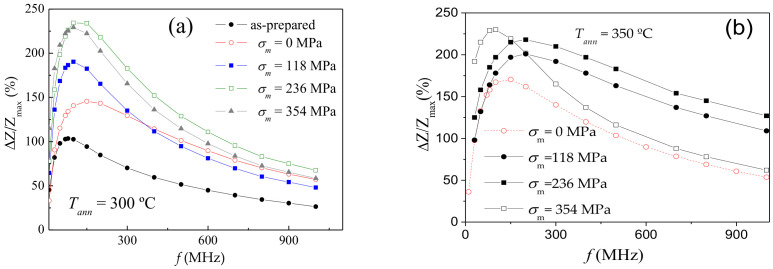
Frequency dependence of maximum GMI ratio, *ΔZ/Z_max_*, in as-prepared and stress-annealed at *T_ann_* = 300 °C (**a**) and *T_ann_* = 350 °C (**b**) Co_69.2_Fe_3.6_Ni_1_B_12.5_Si_11_C_1.2_Mo_1.5_ microwires.

As expected from Figure 4a, further optimization of GMI performance of Co_69.2_Fe_3.6_Ni_1_B_12.5_Si_11_C_1.2_Mo_1.5_ microwires can be achieved upon selection of proper frequency. Frequency dependencies of *ΔZ/Z_max_* for the microwires annealed at *T_ann_* = 300 and *T_ann_* = 350 °C are shown in Figure 6a,b, respectively. For both *T_ann_*, the highest *ΔZ/Z_max_* are observed for intermediate (236 MPa) *σ_m_*-values. The optimal frequency for all stress-annealed Co_69.2_Fe_3.6_Ni_1_B_12.5_Si_11_C_1.2_Mo_1.5_ microwires is shifted to higher *f*-range. Indeed, the highest *ΔZ/Z_max_* –values of as-prepared Co_69.2_Fe_3.6_Ni_1_B_12.5_Si_11_C_1.2_Mo_1.5_ microwire are observed for *f* = 100 MHz, while for stress-annealed Co_69.2_Fe_3.6_Ni_1_B_12.5_Si_11_C_1.2_Mo_1.5_ microwires the highest *ΔZ/Z_max_* –values are observed at 150–200 MHz (see Figure 6). The obtained frequency range (100–200 MHz) in which the highest *ΔZ/Z_max_* –values are observed in studied microwires are within the preferred range for sensors applications: a better signal to noise features are observed for 10–200 MHz [74].

Additionally, appropriate post-processing (stress-annealing at *T_ann_* = 300 °C and *σ_m_* = 236 MPa or at *T_ann_* = 350 °C *σ_m_* = 354 MPa) allows remarkable GMI ratio improvement (from about 100% up to about 240%).

**Figure 7:**
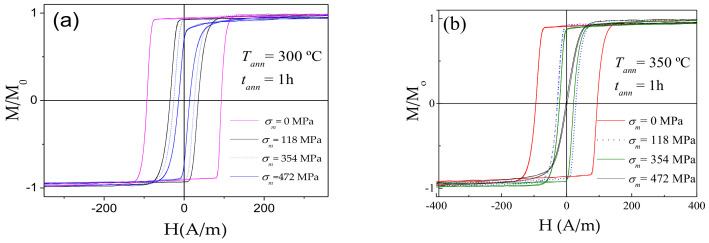
Hysteresis loops of Co_69.2_Fe_3.6_Ni_1_B_12.5_Si_11_C_1.2_Mo_1.5_ microwires annealed at *T_ann_* = 300 °C (**a**) and *T_ann_* = 350 °C (**b**) for different *σ_m_*.

As can be appreciated from Figure 7, the hysteresis loops remain rectangular shape after stress-annealing at given annealing temperatures (300 °C and 350 °C) if *σ_m_* < 472 MPa. The common feature of all stress-annealed samples is lower coercivity, *H_c_*. For both selected *T_ann_*, the hysteresis loops became inclined at *σ_m_* = 472 MPa.

**Figure 8:**
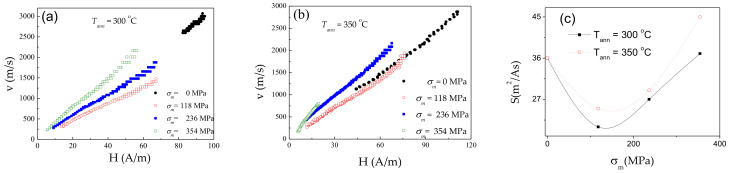
*v(H)* dependencies of annealed at *T_ann_* = 300 °C (**a**) and 350 °C (**b**) Co_69.2_Fe_3.6_Ni_1_B_12.5_Si_11_C_1.2_Mo_1.5_ microwires and *S*(*σ_m_*) dependencies for the same microwires (**c**).

Accordingly, the DW dynamics has been studied in Co_69.2_Fe_3.6_Ni_1_B_12.5_Si_11_C_1.2_Mo_1.5_ microwires annealed at 300 °C and 350 °C for *σ_m_* < 472 MPa (see Figure 8a,b). The difference in *v(H)* dependencies is that the magnetic field range of single DW propagation is shifted to low–field region (see Figure 8a,b). Therefore, maximum DW velocity values are generally below 2.5 km/s. However, DW mobility remarkably depends on *σ_m_*: for the highest *σ_m_* the highest *S*-values are achieved (see Figure 8c).

Accordingly, aforementioned results on effect of post-processing on magnetic properties of Co_69.2_Fe_3.6_Ni_1_B_12.5_Si_11_C_1.2_Mo_1.5_ microwires provide a route allowing to obtain a unique combination of magnetic properties (fast single DW dynamics and high GMI ratio) in the studied microwire. In fact, the transformation of linear hysteresis loop into a rectangle, and hence, the coexistence of fast DW propagation and GMI effect has been reported in various Co-rich microwires [75,76,77]. Evidently, post-processing (annealing) conditions for Co-rich microwires with different compositions can be distinct. Thus, annealed and stress-annealed Co_69.2_Fe_4.1_B_11.8_Si_13.8_C_1.1_ or Co_50.69_Fe_8.13_Ni_17.55_B_13.29_Si_10.34_ glass-coated microwires can also present both high GMI ratio and fast propagation of single DW [75,76,77]. However, the annealing conditions for Co_69.2_Fe_4.1_B_11.8_Si_13.8_C_1.1_ and Co_50.69_Fe_8.13_Ni_17.55_B_13.29_Si_10.34_ glass-coated microwires are different.

Although Co-rich microwires generally present best magnetic softness, Co belongs to critical raw materials [78]. Accordingly, Co-rich microwires are more expensive and the insecure Co supply might be critical for the development of new technologies. Therefore, less expensive Fe-based microwires are preferable for massive applications. However, as-prepared Fe-rich microwires present poor GMI effect [58,59,61]. Accordingly, recently certain attention has been paid to optimization of GMI effect in Fe-rich microwires [59,61].

### 3.2. Tuning of GMI Effect and DW Dynamics in Fe-Rich Microwires

Magnetic softening and enhanced GMI effect have been observed in nanocrystalline Fe-rich magnetic microwires prepared by devitrification of amorphous FeSiBCuNb precursor [13,58,79]. However, devitrification is usually accompanied by the brittleness of microwires, which severely limits the applications possibility. Accordingly, alternative post-processing approaches enabling to enhance the GMI effect retaining the amorphous structure have been explored [58,61,62,63].

**Figure 9:**
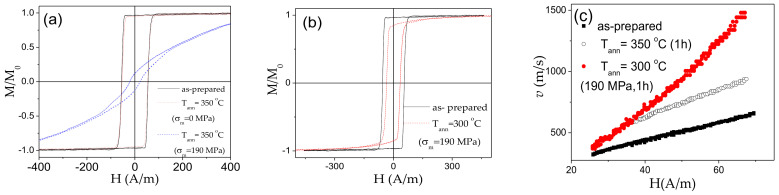
Hysteresis loops of as-prepared, annealed and stress- annealed at *T_ann_* = 350 °C Fe_75_B_9_Si_12_C_4_ microwires (**a**), hysteresis loops of as-prepared and stress-annealed at 300 °C Fe_75_B_9_Si_12_C_4_ microwires (**b**) and *v(H)* dependencies of as-prepared, annealed at *T_ann_* = 350 °C and stress-annealed at *T_ann_* = 300 °C Fe_75_B_9_Si_12_C_4_ microwires (**c**).

In contrast to Co-rich microwires, hysteresis loops of stress-annealed (even for low *σ_m_* = 190 MPa) Fe_75_B_9_Si_12_C_4_ microwires rapidly transform into inclined (see Figure 9a). However, the hysteresis loop of Fe_75_B_9_Si_12_C_4_ microwire annealed at the same *T_ann_* (without stress) retains its rectangular shape (see Figure 9a). The rectangular hysteresis loop has been retained only after stress-annealing at *T_ann_* = 300 °C (190 MPa, 1 h) (see Figure 9b). Accordingly, single DW propagation is observed in both as-prepared, annealed (*T_ann_* = 350 °C) and stress-annealed (*T_ann_* = 300 °C, *σ_m_* = 190 MPa) microwires (see Figure 9c). In spite of almost the same hysteresis loops and similarly to other Fe-rich microwires [36,59,62], a remarkable increase in DW velocity (from 0.65 up to almost 1 km/s at H = 68 A/m) and DW mobility (from 7.3 up to 11.2 m^2^/As) is observed upon annealing. More remarkable increase in DW velocity (up to almost 1.5 km/s at H = 68 A/m) and DW mobility (up to 27 m^2^/As) is observed in stress-annealed Fe_75_B_9_Si_12_C_4_ microwire.

**Figure 10:**
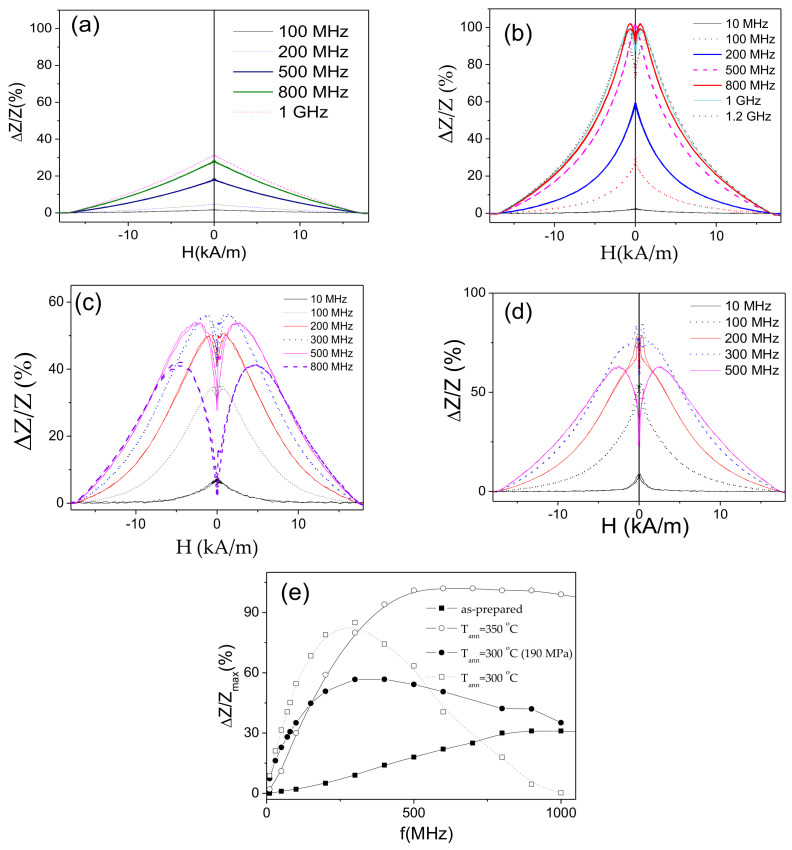
*ΔZ/Z(H)* dependencies of as-prepared (**a**); annealed (**b**) and stress-annealed at *σ_m_* = 190 MPa (**c**) at *T_ann_* = 350 °C; annealed at *T_ann_* = 300 °C for *σ_m_* = 0 MPa (**d**) and *ΔZ/Z_m_(f)* dependencies of as-prepared, annealed at *T_ann_* = 350 °C and stress- annealed at *T_ann_* = 300 °C (190 MPa) (**e**) Fe_75_B_9_Si_12_C_4_ microwires.

The influence of annealing on *ΔZ/Z (H)* dependencies and *ΔZ/Z_max_* –values is shown in Figure 10. As can be observed, annealing allows considerable increase in *ΔZ/Z_max_* –values in the whole *f*-range (see Figure 10e). Some increase in *ΔZ/Z_max_* –values is also observed for stress-annealed sample. However, in the present case (when the hysteresis loops are still rectangular), annealing is more efficient, in terms of optimizing the GMI effect. Additionally, for *f* ≥ 500 MHz and *f* ≥ 100 MHz *ΔZ/Z(H)* dependencies of annealed and stress-annealed Fe_75_B_9_Si_12_C_4_ samples, respectively, exhibit double-peak shape typical for weak transverse magnetic anisotropy (see Figure 10b–d). Such frequency effect on *ΔZ/Z*(*H*) dependence shape was recently discussed, considering dependence of skin depth, δ, on frequency as well as radial distribution of magnetic anisotropy that can be related either to transverse magnetic anisotropy in the surface layer or the interfacial layer between the metallic nucleus and the glass-coating [62,80].

**Figure 11:**
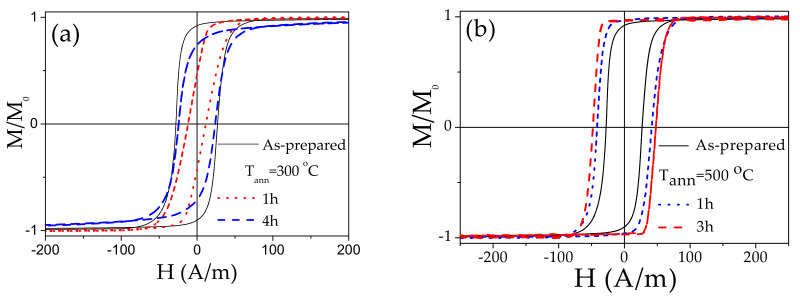
Hysteresis loops of as-prepared and annealed at 300 °C (**a**) and 550 °C (**b**) Fe_71.7_B_13.4_Si_11_Nb_3_Ni_0.9_ glass-coated microwires. Adapted from ref. [45].

In the case of other Fe-rich microwire (Fe_71.7_B_13.4_Si_11_Nb_3_Ni_0.9_) with thicker diameter the rectangular hysteresis loops are observed in as-prepared and annealed at 550 °C samples (see Figure 11). However, the hysteresis loops of Fe_71.7_B_13.4_Si_11_Nb_3_Ni_0.9_ microwires annealed at intermediate temperature (300 °C) are not perfectly rectangular (see Figure 11a).

**Figure 12:**
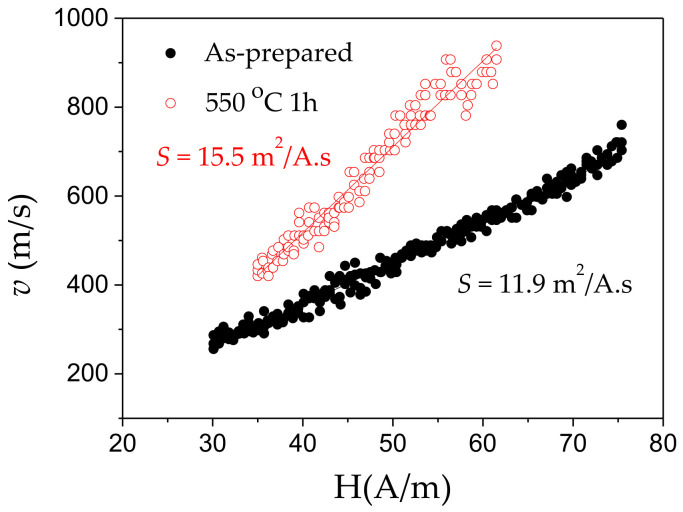
*v(H)* dependencies of as-prepared and annealed (550 °C 1 h) Fe_71.7_B_13.4_Si_11_Nb_3_Ni_0.9_ microwires. Reproduced from Ref. [45] with permission.

As observed, beginning of crystallization process is observed after annealing at 550 °C for 3 h [45]. Therefore, Fe_71.7_B_13.4_Si_11_Nb_3_Ni_0.9_ microwires annealed at *T_ann_* = 550 °C (1 h) are still amorphous and hence present good mechanical properties. Accordingly, single DW dynamics has been observed in as-prepared and annealed at *T_ann_* = 550 °C (1 h) Fe_71.7_B_13.4_Si_11_Nb_3_Ni_0.9_ microwires (see Figure 12).

As can be appreciated from Figure 12, even as-prepared microwire presents relatively high DW velocity with maximum DW velocity of about 700 m/s.

*S*-values, experimentally evaluated from the *v*(H) dependence using the expression (3) provide *S*≈11.9 m^2^/As, being higher than that usually observed for as-prepared Fe-rich microwires (compare with *S*≈7.3 m^2^/As evaluated from Figure 9). As can be observed from Figure 12, annealing allows further improvement of DW velocity (up to 1 km/s) and mobility (up to *S*≈15.5 m^2^/As).

**Figure 13:**
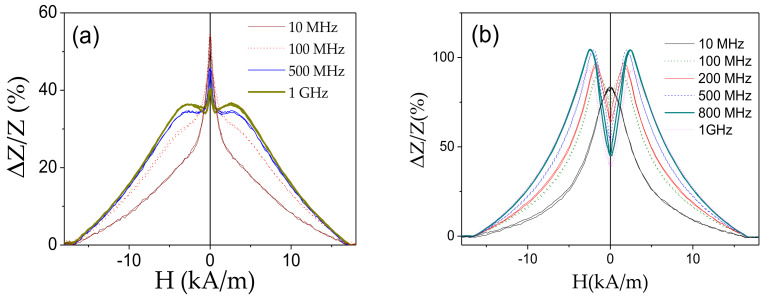
*ΔZ/Z(H)* dependencies of as-prepared; and (**a**) annealed at 550 °C (1 h) (**b**) Fe_71.7_B_13.4_Si_11_Nb_3_Ni_0.9_ microwires measured at different frequencies. Figure 12a is adapted from ref. [62].

Annealing of Fe_71.7_B_13.4_Si_11_Nb_3_Ni_0.9_ microwire also allows increasing in *ΔZ/Z_max_* –values from 55% up to 105% (see Figure 13). Additionally, *ΔZ/Z*(*H*) dependence of as-prepared Fe_71.7_B_13.4_Si_11_Nb_3_Ni_0.9_ microwire has irregular shape that recently has been attributed to the superposition of the double-peak *ΔZ/Z(H)* dependence from the region with transverse magnetic anisotropy and the single-peak dependence from the wire portion with axial magnetic anisotropy [62]. After annealing, *ΔZ/Z*(*H*) dependence presents more regular double-peak shape for *f* ≥100 MHz (see Figure 13b).

**Figure 14:**
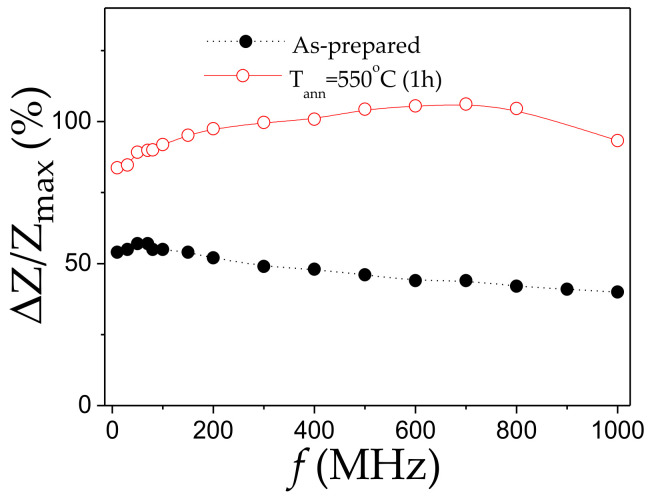
Frequency dependence of maximum GMI ratio of as-prepared and annealed at 550 °C (1 h) Fe_71.7_B_13.4_Si_11_Nb_3_Ni_0.9_ microwires.

As can be observed from *ΔZ/Z_max_* (*f*) dependence (see Figure 14), superior *ΔZ/Z_max_* -values are observed in annealed Fe_71.7_B_13.4_Si_11_Nb_3_Ni_0.9_ microwires in the whole frequency range.

The relatively high GMI ratio of annealed Fe-rich microwires, with rectangular hysteresis loops, appears unexpected. Although it is generally believed that high circumferential permeability is the most relevant parameter for achieving high GMI effect, several publications indicate the importance of the damping parameter [70,80]. Consequently, the increase in the GMI ratio observed in annealed Fe-rich microwires (see Figure 10, Figure 13 and Figure 14) may be due to the impact of annealing on the damping parameter, evidenced by the significant increase in DW mobility after annealing (see Figure 9 and Figure 12).

Among the factors that can affect the GMI performance are the surface conditions that can affect the surface domain structure [81]. Such domain structures is affected by the surface roughness [81]. On the other hand, the surface irregularities and the magnetoelastic contribution in DW pinning are identified among the factors affecting magnetic softness of amorphous materials [82]. In the present case, the magnetoelastic contribution related to strong internal stresses originated by the presence of glass-coating is even more relevant than for conventional amorphous materials [46,47,48].

On the other hand, the microwires inhomogeneities can substantially affect the DW dynamics, as observed by comparing the local nucleation field distribution and the defects distribution along the microwires [83]. The origin of the defects has been discussed elsewhere [36,83], considering the inhomogeneous stresses distribution, shape irregularities, oxides, etc.

Considering that all the properties have been measured on the same samples (i.e., all the measurements have been performed in as-prepared sample and then, the same samples have been post-processed) we consider the magnetoelastic contribution, as well as induced magnetic anisotropy are the most relevant.

The above-described post-processing is essentially relevant for obtaining the unique combination of fast and single DW propagation and GMI effect in the same microwire. Such combination of properties has been obtained in annealed or stress-annealed microwires. The main role of annealing is usually associated to internal stresses relaxation. However, in amorphous alloys containing two or more ferromagnetic elements, the DW stabilization due to directional atomic pairs ordering can be relevant [84,85,86]. This is not the case for studied Fe-rich microwires (Fe_75_B_9_Si_12_C_4_). However, such induced magnetic anisotropy, originated by a preferred magnetization direction during the annealing, can be considered for Co-rich (Co_69.2_Fe_3.6_Ni_1_B_12.5_Si_11_C_1.2_Mo_1.5_) microwire.

The mechanisms involved in stress-annealing induced anisotropy of amorphous microwires have been discussed considering either back stresses or short range ordering [80,87,88,89,90]. The back stresses give rise to the internal stresses redistribution during the stress-annealing [80,87,90]. Such mechanism can be dominant for microwires with only one ferromagnetic element (Fe_75_B_9_Si_12_C_4_). The short range ordering can be originated either by atomic directional pair ordering (commonly considered for amorphous alloys with two or more ferromagnetic elements, i.e., relevant for Co_69.2_Fe_3.6_Ni_1_B_12.5_Si_11_C_1.2_Mo_1.5_) or by topological short range ordering [80,87,88,89]. The latter can be, in principle, observed in both kinds of studied microwires (Fe and Co-rich). The contribution of topological short range ordering (also known as structural anisotropy) was recently linked to partially irreversible stress-annealing induced anisotropy in Fe-rich microwires [80].

As observed above (see Figure 7 and Figure 9), transverse magnetic anisotropy can be induced in both Fe-rich and Co-rich microwires. However, for Co-rich microwires annealed at the same *T_ann_* higher applied stresses are required to observe substantial transverse magnetic anisotropy.

It is worth mentioning, that the atomic directional pair ordering and related DW stabilization can be relevant if the annealing is performed at temperatures below Curie temperature (between 370 and 415 °C for the studied compositions) [63,64].

The latter Fe-rich (Fe_71.7_B_13.4_Si_11_Nb_3_Ni_0.9_) microwires with the thickest diameter are the most appropriate for the MOKE studies (usually used for studies of planar samples) because of the lowest curvature.

### 3.3. MOKE Experiments and Results

The MOKE experiments were motivated by the importance of the surface area of the microwire in the GMI effect. At the same time, the shape of the surface hysteresis curves and the images of the surface domain structures are the key aspects for the understanding of the origin of large Barkhausen jump.

**Figure 15:**
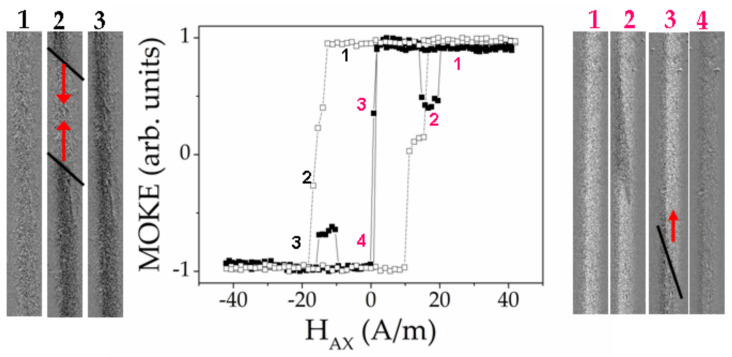
MOKE hysteresis loop (black points) and images of surface domain structure (marked by red numbers) obtained in sample №3 annealed with tension stress. MOKE hysteresis loop (white points) and images of surface domain structure (marked by black numbers) obtained in sample 3 annealed with tension stress in the presence of the torsion of 2 π rad m^−1^.

First, we have studied the Fe_71.7_B_13.4_Si_11_Nb_3_Ni_0.9_ microwires (stress-annealed sample № 3 (curve with black points) and stress-annealed sample № 3 in the presence of torsion stress (curve with white points), see Table 1). Figure 15 presents two MOKE hysteresis loops and the series of the characteristic images of the surface domain structure. 

For the stress-annealed sample № 3, the hysteresis loop contains two characteristic local dips of the MOKE signal. Furthermore, the sharp jump of the signal is observed. Images marked in red numbers №1 and №4, show two mono-domains corresponding to two saturation states. The image №2 is the inclined wedged-like domain, demonstrating the formation of the spiral domain structure [69]. In the image № 3 the moment was fixed when the inclined solitary DW moved quickly along the microwire.

Therefore, we could select two separate processes during the magnetization reversal: Formation of the spiral domain and the quick motion of the elliptic DW. As it is known [91], the formation and transformation of the surface domain structure plays the essential role in the microwire susceptibility, which in turn is the key parameter of the GMI effect. In particular, the DW length is directly related with the susceptibility. Earlier [91,92] we have demonstrated the influence of the spiral domain structure on the dynamic properties of the magnetic microwire. The matter is that the length of the spiral DW is very long and it is limited only by the microwire length. It permits us to consider the spiral domain structure observed in the microwires as a potential candidate for the GMI based sensors.

The second process is the quick inclined elliptic DW running through the surface of the microwire. The black line in the image №3 marks the position of the solitary inclined elliptic DW. The red arrow shows the direction of the DW displacement. This process has a direct relation with the magnetic bistability, precisely “helical” bistability, which is realized in the large Barkhausen jump between two helical magnetic structures [93]. Thus, demonstrating the two mentioned effects, the studied Fe_71.7_B_13.4_Si_11_Nb_3_Ni_0.9_ microwire could be simultaneously used in magnetic sensors based on both GMI and large Barkhausen jump effects.

The application of torsion stress during the MOKE experiment drastically changes the shape of the hysteresis loop and images of the domain structure. The process of the surface magnetization reversal becomes simpler. The torsion stress suppresses the formation of the spiral structure, while supporting the elliptic domain structure [91]. The curve with white points in the Figure 15 presents the MOKE hysteresis loop obtained in the sample № 3 in the presence of torsion stress of 2 π rad m^−1^. The images of the domain structures marked by the black numbers correspond to this hysteresis loop. In this case the shape of the MOKE hysteresis loop is almost rectangular. It is not the clear Barkhausen Jump, but the DW motion could be recognized.

Two inclined domain walls marked by the black lines, get into the field of view from “up” and “down”. The DW motion is not very regular that is reflected in the hysteresis loop. This can be attributed to the distribution of the defects affecting the DW motion. The observed domain structure was qualified as elliptic type of magnetic structure.

At the second stage of the MOKE study we have investigated Co-rich microwires № 5 with the same purpose of determination of the coexistence of different types of the domain structure.

**Figure 16:**
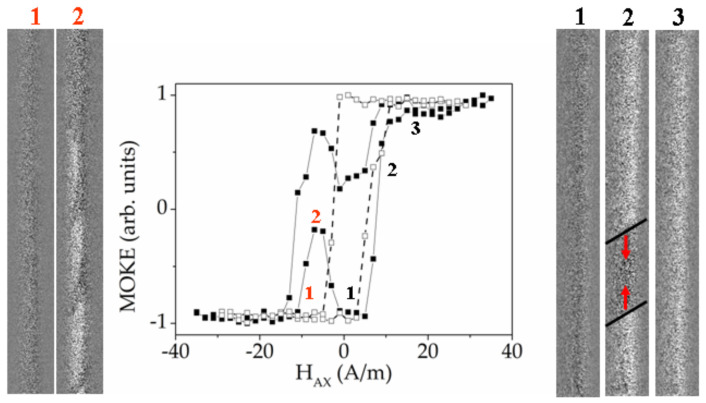
MOKE hysteresis loops and images of surface domain structure obtained in annealed sample №5 (black points) and as-prepared sample №5 in the presence of torsion stress of 1 π rad m^−1^ (white points).

Figure 16 presents the MOKE hysteresis loops and the images of the domain structures obtained in annealed sample №5 (black points) and as-prepared sample №5 in the presence of torsion stress of 1 π rad m^−1^ (white points). The MOKE hysteresis loop obtained in annealed sample contains the specific local dips partially similar to the dips observed in the Figure 15 and related to the formation of spiral magnetic structure. Here, we also observe the formation of this type of domain structure, which is really realized in a different scenario. Although, it looks like the multi-domain structure, in reality we observe the solitary spiral domain which moves along the microwire enfolding it around (left image 2 marked in red). Generally, this type of the dips is related to the irregular process of the change of the period of the spiral domain structure. We note that the fast DW motion, related to Barkhausen jump, was not observed during this experiment.

As in the case of Fe-rich microwire presented above, the application of torsion stress suppresses the spiral magnetic structure and supports the elliptic domain structure. The basic difference in these types of domain structure is the length of the DW [92]. The length of the elliptic DW is much shorter than that of the spiral DW and it is determined by the length of the inclined ellipse.

**Figure 17:**
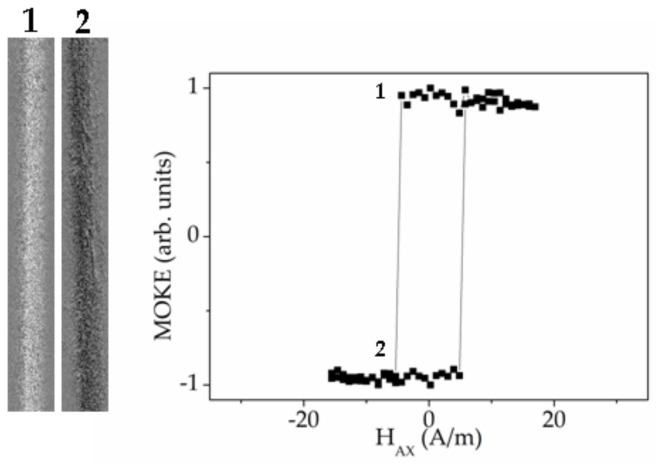
MOKE hysteresis loop and images of surface domain structure obtained in as-prepared sample №5 in the presence of torsion stress of 2 π rad m−1.

For the case of the as-prepared sample №5 in the presence of torsion stress of 1 π rad m^−1^ (white points and right images marked in black), the image №3 demonstrates the moment of the oppositely directed motion of two elliptic DW. The possibility of identifying the DW motion during the sharp jump of the magnetization indicates that the single Barkhausen jump has not yet reached. The further increase of the torsion stress leads to the realization of single Barkhausen jump (Figure 17). According to the nature of the single Barkhausen jump only the images of the domain structures corresponding to the mono-domain states before and after the jump could be fixed. Namely these images 1 and 2 are demonstrated in the Figure 17.

Here, we present the video (Video 1, reproduced from Ref. [92] with permission) demonstrating the magnetization reversal process corresponding to the as-prepared sample №5 in the presence of torsion stress of 1 π rad m^−1^. The time duration of the video is about 11 sec. The linearly changed magnetic field directed along the wire axis was applied. It was ranged from −2 A/m to −5 A/m during the experiment. Black and grey colours of the domains correspond to axial directions of the magnetization. The black domain enters to the field of microscopy view at the moment, corresponding to the sixth second from the beginning. It means that the elliptic inclined DW separates to oppositely magnetized domains. The second inclined DW appears at the bottom of the video frame at the moment corresponding to the eighth second. Two walls move towards each other until the gray domain disappears completely.

**Figure 18:**
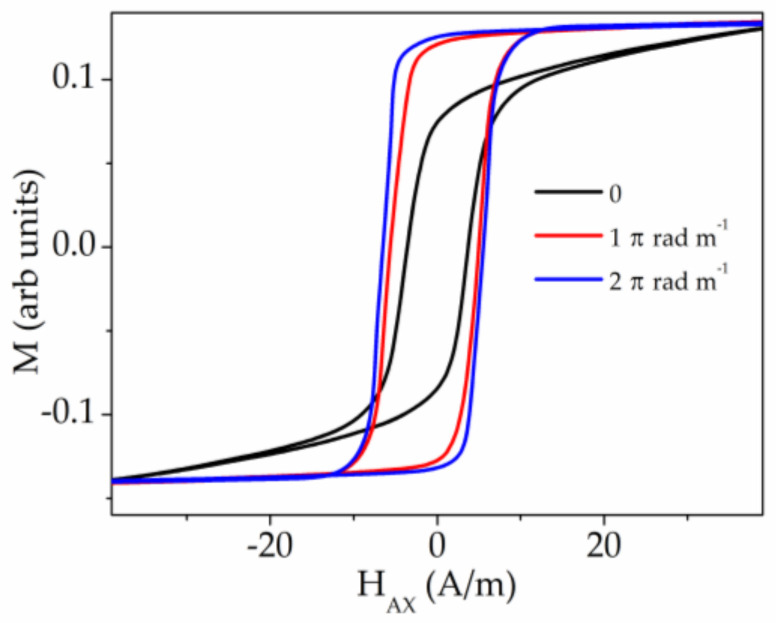
Magnetic hysteresis loops obtained in as-prepared sample №5 in the presence of torsion stress.

The additional confirmation of our concept is the magnetic hysteresis loops presented in the Figure 18. These hysteresis loops, unlike MOKE, contain the information about the magnetization reversal in the whole sample, not just in its surface. According with the conclusions from our MOKE experiment, the torsion stress induces the Barkhausen jump. At the same time, the process of the hysteresis loop transformation passes through the stage when, becoming more rectangular, the curve has not yet reached the bistability (red line in Figure 18). This phase of the stress induced transformation corresponds to the MOKE hysteresis loop presented in the Figure 16 (white points).

A comparative analysis of MOKE and bulk hysteresis loops allows us to consider the studied Co-rich and Fe-rich microwires as very promising candidates for a basic element in various types of magnetic sensors. We were able to control reversible transitions between different types of magnetic structures that exist both on the surface and inside the microwire. A predictable selection of magnetic structure can serve as a good basis for real-time switching between different types of magnetic sensors operating on the same microwires.

## 4. Conclusions

We studied the influence of various post-processing on the magnetic properties, DW dynamics and GMI effect of Fe- and Co-rich microwires with aim to identify the routes to obtain microwires with unique combination of magnetic properties allowing fast and single DW propagation and GMI effect to be observed in the same microwire.

In several Co-and Fe-rich microwires with rectangular hysteresis loops, we observed a high GMI effect. For optimization of DW dynamics and GMI effect we used conventional furnace annealing and stress-annealing. By modifying the annealing conditions, we have identified the appropriate regimes allowing achievement of the highest GMI ratio in microwires with rectangular hysteresis loops. Irregular magnetic-field-dependence of GMI ratio, observed in stress-annealed Fe-rich microwires, were discussed, in terms of the contribution of both inner axially magnetized core and outer shell with transverse magnetic anisotropy. The observed experimental dependencies are discussed considering radial distribution of magnetic anisotropy and the correlation of the GMI effect and DW dynamics, and with bulk and surface magnetization processes.

The MOKE experiments performed in both Fe- and Co-rich microwires provide the information about magnetic structure in the surface area of the microwires that allowed us to focus mainly on the magnetic behaviour in the outer shell of the microwires. We have demonstrated the existence of the spiral helical structure in two studied types of microwires. The presence of such a structure is a favourable feature for use these microwires in GMI based sensors. At the same time, torsion mechanical stress induces helical bistability in the same microwires, making them suitable for sensors based on the large Barkhausen jump.

## Figures and Tables

**Table 1:** Compositions and geometry of studied glass-coated microwires.

Sample №	Composition	Metallic Nucleus Diameter, d (μm)	Total Diameter, D (μm)	Ratio *ρ* = d/D	Magnetostriction Coefficient, λ_s_ × 10^−6^	Crystallization Temperature, *T_c_* (°C)
1	Fe_75_B_9_Si_12_C_4_	15.2	17.2	0.88	38	522
2	Co_69.2_Fe_3.6_Ni_1_B_12.5_Si_11_C_1.2_Mo_1.5_	22.8	23.2	0.98	−1	553
3	Fe_71.7_B_13.4_Si_11_Nb_3_Ni_0.9_	103	158	0.65	35	570
4	Co_69.2_Fe_4.1_B_11.8_Si_13.8_C_1.1_	25.6	30.2	0.85	−0.03	507
5	Co_64,04_Fe_5,71_B_15,88_Si_10,94_Cr_3.4_Ni_0,3_	94	126	0.75	2	562
